# Validating and applying a multidimensional scale to measure public security police stressors in China

**DOI:** 10.3389/fpubh.2026.1932210

**Published:** 2026-09-03

**Authors:** Jue Deng, Jianping Ma, Yuanhua Ou, Xianghui Lai, Zhenqiang Li, Xiaoli Zhu

**Affiliations:** 1 Cognitive Neuroscience and Abnormal Psychology Laboratory, Department of Penalty Execution, Fujian Police College, Fuzhou, China; 2 Fujian Key Laboratory of Applied Cognition and Personality, Minnan Normal University, Zhangzhou, China; 3 Department of Basic Courses, Fujian Police College, Fuzhou, China; 4 Department of Education and Training, Fujian Provincial Department of Public Security, Fuzhou, China; 5 Narcotics Control Research Center of Southeastern China, Fujian Police College, Fuzhou, China

**Keywords:** China, police officer, public security, stressors, validation

## Abstract

**Background:**

Policing is a highly stressful occupation, with considerable implications for officers' mental health and wellbeing. Despite increasing research on police stress, there remains a critical need for culturally relevant and up-to-date measurement tools, particularly in Eastern countries like China. This study aimed to develop and validate a revised version of the Police Stress Scale (PSS)—the Revised Chinese Public Security Police Stressors Scale (CPSPSS-R)—a tool assessing stressors affecting Chinese public security police officers.

**Methods:**

The CPSPSS-R was developed using qualitative interviews with 30 police officers and a subsequent psychometric evaluation involving 1,194 in-service officers. The final scale comprised 37 items across four dimensions: excessive workload, lack of skill, organizational issues, and work–family conflict.

**Results:**

The CPSPSS-R demonstrated excellent reliability and validity, with confirmatory factor analysis supporting the four-factor structure (χ^2^/df = 3.76, TLI = 0.916, CFI = 0.922, SRMR = 0.048, RMSEA = 0.070). The scale also found significant correlations between stressors and demographic variables, including age, gender, rank, and educational level.

**Conclusions:**

These findings highlight the demand for differentiated, subgroup-specific stress interventions for police officers. As a psychometrically sound and culturally appropriate scale for evaluating stressors experienced by Chinese public security police officers, the CPSPSS-R yields evidence to enhance police management systems and advance police officers' overall wellbeing. More importantly, this scale establishes a vital assessment framework to detect impairments in police officers' psychological adaptation to occupational strain, supporting the design of precise psychological intervention strategies that maintain their long-term mental well-being under constant work-related stress.

## 1 Introduction

Policing is widely recognized as one of the most demanding and stressful professions, given the inherently challenging nature of police work and the high societal expectations placed on officers (1–3). For frontline public security police officers contending with constant emergency hazards, public surveillance and chronic workload overload, unrelieved long-term occupational stressors gradually erode their cognitive and emotional adaptive capacities, sequentially diminishing psychological resilience, life satisfaction, and mental wellbeing (2, 4). Law enforcement administrators are increasingly acknowledging the profound impact of stress on officers, embracing a new era of stress awareness (5). Since foundational research on police work stress in the 1950s (6), studies on police officers' stress and mental wellbeing have steadily expanded. Nevertheless, three critical issues remain unresolved in the global effort to improve stress management and wellbeing among police officers.

First, most existing studies focus on reactive responses to police officers' stress rather than on preventing stressors (5). Stressors are commonly defined as job-related and organizational factors that have the potential to cause stress (7–9). Several studies have investigated perceived stress levels and their psychological effects (10–15). Nevertheless, organizations often choose to implement reactive interventions such as counseling, stress management programs, and stress inoculation training (1, 16). Although these approaches are greatly valuable, they inherently allow stress to persist by addressing its consequences rather than the causes (17). Furthermore, health and wellness initiatives often focus on traumatic stressors, neglecting routine and avoidable stressors. From a preventative and early intervention perspective, greater emphasis should be placed on reducing stressors and preemptively mitigating stress before it leads to harm.

Second, most existing research on police stress is based in Western contexts, leading to a limited understanding of how cultural and systemic factors shape the experiences and determinants of police stress (3, 10, 18). China's distinct social, cultural, and operational contexts—such as severe understaffing relative to its massive population, strict gun control regulations, and a hierarchical organizational structure in the police force that demands unquestioned obedience—create a highly stressful work environment for police officers (19, 20). These challenges often result in excessive overtime as officers juggle emergencies, threats of violence, and extensive administrative duties (21–23). According to a press conference of the (24), 1482 police and auxiliary officers died in the line of duty, and approximately 25,000 were injured over the past 5 years.

It should be highlighted that large-scale violent disturbances are extremely uncommon amid the stable social environment of mainland China, so very few police line-of-duty fatalities stem from such incidents. As documented in publicly released official reports (25, 26), overwork-induced sudden death constitutes the leading cause of fatalities for officers who perish in the line of duty. A breakdown of specific mortality causes reveals the following statistics (26): sudden cardiac arrest and other acute medical emergencies brought on by chronic overtime and persistent fatigue account for over half of all casualties; traffic accidents occurring during law enforcement duties rank as the second most prevalent cause, responsible for 26.3% of line-of-duty fatalities; deaths resulting from violent attacks while confronting criminal suspects make up 5.2% of total line-of-duty fatalities. Sustained exposure to occupational risks, combined with overwhelming workloads and erratic shift rosters, creates a demanding working environment in which job stressors build up incrementally. This reality further underscores the critical need for research examining work-related stress among Chinese public security police officers.

Third, despite an increasing number of studies on occupational stress and mental health in law enforcement, there is a lack of suitable measurement instruments specifically designed to evaluate police work stressors (3), particularly for Chinese officers. When studying stress in police officers, researchers often rely on tools developed for other professional groups, which fail to capture the unique demands of police work (11, 12, 27–30). Currently, only two instruments have been developed to assess stressors among Chinese police officers (4, 31), both of which have limitations and lack relevance to the realities of modern Chinese policing.

The first of the two scales mentioned above is the Police Stress Scale (PSS; 31). It was the first scale designed to measure stressors among police officers in mainland China. While groundbreaking, the PSS has two key limitations: (1) Over a decade has passed since its development, rendering some items outdated and no longer reflective of current police work; (2) The PSS lacks clarity regarding its applicability across different police classifications in China, where police forces are divided into six distinct departments. These departments cover a wide range of responsibilities, including public safety, prison guarding, traffic management, discipline and correction, legal education, household registration, and immigration control, among others (32). Each department presents unique challenges, but officers serving in public safety organizations face particularly high levels of stress and burnout (33). This is due to their exposure to greater dangers, uncertainties, and unpredictability, as well as the demanding requirement of constantly being on call to respond to emergencies, compared to other departments (34). As per data released by the Committee of Political and Legal Affairs of the CPC Central Committee in 35, Public security officers experience the highest number of casualties among all police forces. The number of public security officers killed in the line of duty is at least 7.2 times higher than that of other departments (35). Given these risks, a targeted measure for assessing stressors among public security officers is crucial.

The second of the two scales was developed to address concerns with the PSS. This was the Occupational Stressors Scale in Policeman of Public Security Organizations (OSSPPSO; 4). Unlike the PSS, the OSSPPSO is specifically designed for use with public security officers. However, it also has two limitations: (1) Although the OSSPPSO contains fewer items (22 items) compared to the PSS (43 items), there is considerable overlap in item content. This redundancy is an inherent challenge when developing measures for specific occupational groups. (2) The OSSPPSO lacks a crucial component of police stressors—the work-home interface (36, 37). According to the work-home resources model [(38, 39); Yang et al., 2023], individuals have limited resources and an increase in resource consumption in one area (e.g., work) inevitably reduces resources available for other areas (e.g., family). The resulting resource depletion can lead to negative emotions and work–family conflict. Therefore, a comprehensive police stress scale should include items about the work-home interface.

After comparing the PSS and OSSPPSO (4, 31), we decided to revise the PSS for three reasons: (1) The PSS includes work-home interface stressors, which the OSSPPSO does not. (2) Although the PSS does not specify which police force it is applicable to, its content broadly covers police stressors, including those relevant to public security officers. This makes it well-suited for adaptation as it aligns with our goal of including as many potential stressors as possible. (3) Although the PSS and OSSPPSO share some similar content, the PSS contains more items, offering greater flexibility for revision.

The current study aimed to revise the PSS and develop an updated, more applicable measurement tool for evaluating stressors among public security police officers—the Revised Chinese Public Security Police Stressors Scale (CPSPSS-R). We conducted a psychometric evaluation of the CPSPSS-R, identified and validated key stressors factors among Chinese public security police officers, and applied the revised scale to a phenomenological investigation of police stress.

## 2 Materials and methods

### 2.1 Measuring tools

#### 2.1.1 Police stress scale

The original Police Stress Scale (PSS) and its psychometric evaluation were published in Chinese in 2012 (31). The PSS comprises 43 items across six dimensions—work task (9 items), interpersonal activities (9 items), power struggles (8 items), capability and skills (7 items), negative emotions (5 items), and organizational management (5 items). Examples of items from each dimension are shown in Table 1. For each item, participants first indicate whether they have experienced the stressful event. If the event did not occur, that item is scored zero (“Have not experienced”). If the event did occur, participants rate the impact level of the event, ranging from one (No Impact) to five (Extreme). The internal reliability of the six-dimensional subscales has been found to be acceptable, with Cronbach's alpha coefficients ranging 0.77–0.93, and the split-half reliability of the six subscales ranging 0.74–0.91 (31).

**Table 1:** Factors and item examples of the original PSS.

Factor	Number of items	Item example
Work task	9	Heavy workload; Working overtime
Interpersonal activities	9	Infrequent communication with family due to long work hours
Power struggles	8	High-pressure competition among colleagues
Capability and skills	7	Lack of proficiency in job skills
Negative emotion	5	Monotonous and dull work content causing irritability
Organizational management	5	Frequent inspections, evaluations, and assessments

#### 2.1.2 Revised Chinese public security police stressors scale

The Revised Chinese Public Security Police Stressors Scale (CPSPSS-R) was developed with approval from the corresponding author of the original PSS. Inclusion of the word “revised” in the scale name reflects both its measurement purpose and its specific focus on public security police officers.

To ensure that items were relevant and reflected current challenges, we conducted qualitative interviews with 30 police officers from public security organizations across five regions of mainland China, including urban and rural areas. The participants represented a diverse range of public security police work, such as patrol and community safety, felony investigations, combating criminal drug networks, economic crime detection, rapid intervention and emergency response, smuggling investigations, and so on.

Through in-depth discussions between the authors and participating active police officers, seven new items were added to the original PSS. These items capture emerging stressors faced by public security police officers in recent years, which were not included in the original scale. For example, being live streamed by onlookers during a law enforcement operation and income concerns due to the economic downturn caused by the COVID-19 pandemic. The descriptions of these additional items are presented in Table 2. With the new items, the preliminary CPSPSS-R comprised 50 items total.

**Table 2:** The description of additional items in preliminary CPSPSS-R.

New items to be added
Insufficient income causing financial strain
Disproportionate pay relative to work effort
Family conflict affects the working state
Coping with public scrutiny through photographs or live-streams during law enforcement
Public security threats due to emergency incidents
Criticism or disapproval from colleagues
Fear of public opinion and negative comments

Notably, no predetermined factors were assigned to the new items. This was because the original factor structure of the PSS may not apply to the CPSPSS-R. Therefore, the factor structure of the CPSPSS was assessed in the current study.

#### 2.1.3 Satisfaction with life scale

The Satisfaction with Life Scale [SWLS; (40)] is a unidimensional scale, comprising five items. Responses are rated on a 7-point Likert scale ranging from one (do not agree at all) to seven (very strongly agree), with higher scores indicating greater life satisfaction. The Chinese version of the SWLS has been found to be valid and reliable, with a Cronbach's α coefficient of 0.78 and split-half reliability of 0.70 (41). In our study (*n* = 1,133), Cronbach's α was 0.88, McDonald's ω was 0.89, and Guttman's split-half coefficient was 0.81.

The SWLS was selected as the criterion variable not only for validity testing but also as a core indicator of general psychological wellbeing and successful life adaptation. On the one hand, previous research has shown that higher perceived stress levels are associated with lower life satisfaction (41). On the other hand, life satisfaction serves as a key marker of adaptive psychological functioning (42, 43): officers exposed to severe occupational stressors typically display poorer cross-domain adaptation, as reflected in reduced SWLS scores.

### 2.2 Participants and procedure

Recruitment information was disseminated to all police officers across five jurisdictions in Southeast Mainland China with the assistance of police staff overseeing police administration and officers' mental health. Questionnaires including all measurement instruments were printed beforehand. Each police officer independently decided whether to participate after understanding the questionnaire content and research aims. Via this recruitment strategy, a total of 1,194 active-duty public security police officers were enrolled in the study. To ensure data quality, three postgraduate students specializing in psychometrics were trained to complete the data collection. After screening the collected data, 61 responses were discarded due to deceptive answers or missing data exceeding 10% (44, 45). The final sample size was 1,133, yielding an effective response rate of 94.9%. To evaluate the test–retest reliability, 100 of the police officers were invited to complete the survey after a 30-day interval, and 93 valid responses were collected.

This study was approved by the Ethics Committee of Fujian Police College for research conducted with human subjects on September 15, 2023, and was performed in full compliance with the 1964 Declaration of Helsinki and its later addenda. All participants provided written informed consent before responding to the survey. They were all informed that participation was voluntary and that the survey would be completely anonymous.

### 2.3 Statistical analyses

Statistical analyses were performed using IBM SPSS Statistics 22.0 and Mplus 8.0. The dataset was randomly divided into two groups using SPSS. A total of 558 participant responses were assigned to Sample 1, consisting of 480 males, 58 females, and 20 individuals with an unknown gender. The second set comprised 575 responses and was allocated to Sample 2, which included 480 males, 64 females, and 31 individuals with an unknown gender.

Statistical analysis involved three steps. First, we conducted an item analysis to examine item discrimination. Second, exploratory factor analysis (EFA) and confirmatory factor analysis (CFA) were employed to inspect construct validity. Third, the reliability was assessed using internal consistency coefficients (Cronbach's α and McDonald's ω), split-half reliability, and test-retest reliability. We also assessed criterion validity. Data from Sample 1 was used for item analysis and EFA, whereas Sample 2 data was used for CFA, reliability, and validity. Further, the full sample was used to examine relationships between stressors and demographic variables such as age, educational level, and police rank.

## 3 Results

### 3.1 Demographic characteristics

Participants ages ranged from 21 to 60 years with an average of 38.11 years (SD = 8.59). The average duration of police service was 13.97 years, with the longest tenure spanning 41 years and the shortest lasting less than a year. The sample comprised 960 male participants (84.7%), 122 female participants (10.8%), and 51 participants who did not disclose their gender (4.5%). The ranks of the participants were distributed as follows: 243 constables (21.4%), 371 superintendents (32.7%), and 519 supervisors (45.8%). It should be noted that the rank distribution of police officers in Mainland China does not follow a pyramid structure. At present, the cohort of young police officers with constable rank is smaller than that of middle-aged officers holding police superintendent and supervisor ranks. This structure stems from the following two factors.

One factor is the existing administrative mechanism within police organizations. Mainland China adopts a dual-track system consisting of rank promotion and post promotion. Under the first track, police ranks elevate with extended years of service, accumulated work experience and seniority. For example, a constable may be promoted to police superintendent after three years in service, and this rank is divided into three tiers. Upon reaching sufficient service years, officers with superintendent rank can be further promoted to police supervisor, which also has three grades. This type of promotion is mainly seniority-based, and rank upgrade is accompanied by corresponding salary and welfare increments. The second track refers to post-based promotion, which hinges on whether an officer possesses administrative power in police agencies—in other words, whether they hold a leadership position or take charge of a department. This promotion pathway is independent of rank-based progression. As a result, many police officers in Mainland China qualify for superintendent and supervisor ranks through long-term seniority accumulation but have no administrative leadership roles in police institutions.

The other factor is the long-standing family planning policy in Mainland China. Implemented from the 1980s through the early 2010s, the policy limited most families to one or two children and effectively curbed population growth. Inevitably, this policy has shaped the age structure of the current workforce, resulting in a continuous slowdown in the growth of the young population. This trend is not confined to policing; the overall population of Mainland China is facing a continuous decline in the proportion of young people.

Regarding marital status, 162 participants (14.3%) were unmarried, 892 participants (78.7%) were married, 58 participants (5.1%) had another marital status such as widowed or divorced, and 21 participants (1.9%) did not disclose their marital status.

### 3.2 Item analysis

Participants scoring the top 27% of total scores in Sample 1 were classified as the “high-score” group, and those obtaining the lowest 27% represented a “low-score” group. An independent samples *t*-test was conducted to analyze the discrimination of 50 items. Significant differences were observed between the high- and low-score groups for all items (*t-*values ranging from 13.679 to 28.643, *p* < 0.001), indicating acceptable item discrimination.

Subsequently, we analyzed the correlation between each item score and the CPSPSS-R total score using Pearson's correlation coefficient. An item having an item-total correlation coefficient above 0.4 was considered acceptable (46). The analysis found that five items (items 11, 18, 19, 40, and 45) had *r*-values below 0.4, making them unsatisfactory to include in the scale. The correlations of each item score to CPSPSS-R total score are summarized in Table 3. Unsatisfactory items were excluded from further analysis, leaving 45 items for inclusion in the EFA.

**Table 3:** Pearson's correlations between each item score and total score on CPSPSS-R.

Item	*r*	Item	*r*	Item	*r*	Item	*r*	Item	*r*
1	0.70^***^	11	0.36^**^	21	0.61^***^	31	0.58^***^	41	0.51^***^
2	0.52^***^	12	0.72^***^	22	0.74^***^	32	0.46^***^	42	0.68^***^
3	0.71^***^	13	0.74^***^	23	0.75^***^	33	0.76^***^	43	0.77^***^
4	0.71^***^	14	0.69^***^	24	0.58^***^	34	0.71^***^	44	0.48^***^
5	0.51^***^	15	0.48^***^	25	0.73^***^	35	0.68^***^	45	0.36^**^
6	0.74^***^	16	0.70^***^	26	0.75^***^	36	0.54^***^	46	0.50^***^
7	0.72^***^	17	0.71^***^	27	0.43^**^	37	0.77^***^	47	0.77^***^
8	0.47^***^	18	0.37^**^	28	0.72^***^	38	0.56^***^	48	0.59^***^
9	0.60^***^	19	0.39^**^	29	0.74^***^	39	0.76^***^	49	0.76^***^
10	0.73^***^	20	0.77^***^	30	0.66^***^	40	0.38^**^	50	0.77^***^

*N* = 558; ^**^p < 0.01; ^***^p < 0.001

### 3.3 Exploratory factor analysis

The Kaiser-Meyer-Olkin (KMO) test and Bartlett's test of sphericity confirmed that Sample 1 was suitable for EFA (KMO = 0.977, χ^2^ = 24,360.004, *p* < 0.001, df = 990). After the first factor analysis, four items (items 21, 32, 42 and 46) were removed due to factor loadings below 0.40, and another four items (items 13, 15, 41 and 44) were discarded because they loaded onto multiple dimensions simultaneously. This resulted in a revised scale of 37 items.

After the second factor analysis, we extracted four factors through principal component analysis (PCA) with varimax rotation, accounting for 69.70% of the total variance. The communality of each item ranged from 0.509 to 0.845. The variance explained by each factor was 23.78, 22.63, 15.77, and 7.52%. The eigenvalues of the four factors were 8.798, 8.374, 5.835, and 2.782.

Based on the factor loadings of CPSPSS-R and the dimensions of the original PSS (31), the four factors were identified as: *excessive workload* (12 items), *lack of skill* (13 items), *organizational issues* (8 items), and *work-family conflict* (4 times). All 37 items met the critical factor loading threshold. Details of the four factors (items and loadings) are presented in Table 4 and item examples for each dimension are shown in Table 5.

**Table 4:** Structure matrix and item loadings for the remaining 37 items of CPSPSS-R.

Excessive workload		Lack of skill		Organizational issues		Work-family conflict	
Item	Loading	Item	Loading	Item	Loading	Item	Loading
1	0.778	30	0.704	22	0.676	14	0.534
2	0.815	31	0.658	23	0.661	16	0.705
3	0.714	33	0.644	24	0.764	17	0.717
4	0.790	34	0.720	25	0.814	20	0.543
5	0.734	35	0.742	26	0.797		
6	0.797	36	0.648	27	0.718		
7	0.782	37	0.715	28	0.613		
8	0.688	38	0.647	29	0.587		
9	0.783	39	0.658				
10	0.640	47	0.626				
12	0.580	48	0.658				
43	0.644	49	0.591				
		50	0.668				

**Table 5:** Item examples of 4 factors of the CPSPSS-R.

Factor	Number of items	Item example
Excessive workload	12	Heavy workload and demanding tasks.
Lack of skill	13	New job requirements exceeding current knowledge.
Organizational issues	8	Formalism in leadership management.
Work-family conflict	4	Family's lack of understanding of police work.

The full English version of CPSPSS-R is presented in Supplementary material. The English CPSPSS-R was translated from Chinese by a Chinese–English translator and two psychologists specializing in police mental health research. We encourage further localization and adaptation of the CPSPSS-R by researchers across diverse linguistic and cultural backgrounds.

### 3.4 Confirmatory factor analysis

We employed the maximum likelihood method to analyze the data from Sample 2 using Mplus (version 8.0). The following indices assessed model fit: χ^2^/df values < 5 indicating that the model was acceptable (47); root mean square error of approximation (RMSEA) values < 0.08 and < 0.06, indicating good and very good model fit, respectively (48, 49); standardized root mean square residual (SRMR) values < 0.08 and < 0.05, indicating good and very good model fit, respectively (48, 50); comparative fit index (CFI) and Tucker–Lewis index (TLI) values > 0.9, indicating good model fit (51).

Analyzing the validity of the structural equation model showed that the four-factor model of the CPSPSS-R fit the data well: χ^2^ = 2,324.164, df = 618, TLI = 0.916, CFI = 0.922, Akaike information criterion (AIC) = 48,462.967, Bayesian information criterion (BIC) = 48,992.275, RMSEA = 0.070, SRMR = 0.048.

### 3.5 Construct validity

CPSPSS-R factors were moderately correlated with each other, with correlation coefficients ranging from 0.275 to 0.442 (*p* < 0.01; Table 6). The correlation coefficients between each factors and the CPSPSS-R total score ranged from 0.491 to 0.823 (*p* < 0.001).

**Table 6:** The correlation between the score of each factor, and between the score of factors and the total score of CPSPSS-R.

Factor	CPSPSS-R	1	2	3
1 Excessive workload	0.735^***^			
2 Lack of skill	0.823^***^	0.442^***^		
3 Organizational issues	0.588^***^	0.416^***^	0.331^***^	
4 Work-family conflict	0.491^***^	0.370^***^	0.368^***^	0.275^**^

*N* = 575; ^**^p < 0.01; ^***^p < 0.001

### 3.6 Reliability analysis

Cronbach's α coefficients for each factor in the scale ranged from 0.870 to 0.963, with the coefficient for CPSPSS-R total score being 0.980. McDonald's ω coefficients for each factor in the scale ranged from 0.867 to 0.963, with the coefficient for CPSPSS-R total score being 0.980. The split-half reliability of each factor ranged from 0.889 to 0.955 and for CPSPSS-R total score was 0.916. The test-retest coefficients of each factor ranged from 0.649 to 0.701, with that of the CPSPSS-R total score being 0.728. These results are presented in Table 7.

**Table 7:** Internal consistency reliability and test-retest reliability of CPSPSS-R.

Factors	Cronbach's α coefficients	McDonald's ω coefficients	Split-half reliability	Test-retest coefficients
Excessive workload	0.963	0.963	0.939	0.685^***^
Lack of skill	0.959	0.960	0.926	0.672^***^
Organizational issues	0.962	0.963	0.955	0.701^***^
Work-family conflict	0.870	0.867	0.889	0.649^***^
CPSPSS-R	0.980	0.980	0.916	0.728^***^

*N* = 575 for internal consistency (Cronbach's α, McDonald's ω, split-half reliability); *N* = 93 for test–retest reliability. ^***^p < 0.001

### 3.7 Criterion validity

We considered life satisfaction, measured using the SWLS, as the criterion. To assess the validity, we examined correlations between the CPSPSS-R total score, scores of each CPSPSS-R factor, and the score of SWLS. The CPSPSS-R total score and the score of each factor had a significantly negative correlation with the score of SWLS (Table 8).

**Table 8:** The correlation between the total score of CPSPSS-R/the score of each factor and the score of SWLS.

Variables	Excessive workload	Lack of skill	Organizational issues	Work-family conflict	CPSPSS-R
SWLS	−0.390^***^	−0.356^***^	−0.325^***^	−0.426^***^	−0.407^***^

*N* = 575; ^***^p < 0.001

### 3.8 CPSPSS-R factors and demographic variables

To further investigate the stressors affecting public security officers, we examined correlations among CPSPSS-R stressors, age, duration of police service, and educational level. We also explored difference between genders and among ranks.

CPSPSS-R total score and the score of each factor showed a negative correlation with the age and the length of police service. However, education level was positively correlated with the stressors. Although statistically significant, these correlations are weak (Table 9).

**Table 9:** Correlations between age and length of police service and the scores of CPSPSS-R and subscales.

Variables	Excessive workload	Lack of skill	Organizational issues	Work-family conflict	CPSPSS-R
Age	−0.184^**^	−0.113^**^	−0.092^**^	−0.125^**^	−0.151^**^
Police service length	−0.159^**^	−0.120^**^	−0.095^**^	−0.127^**^	−0.144^**^
Educational level	0.109^**^	0.087^**^	0.107^**^	0.077^**^	0.109^**^

*N* = 1133; ^**^p < 0.01

**Figure 1:**
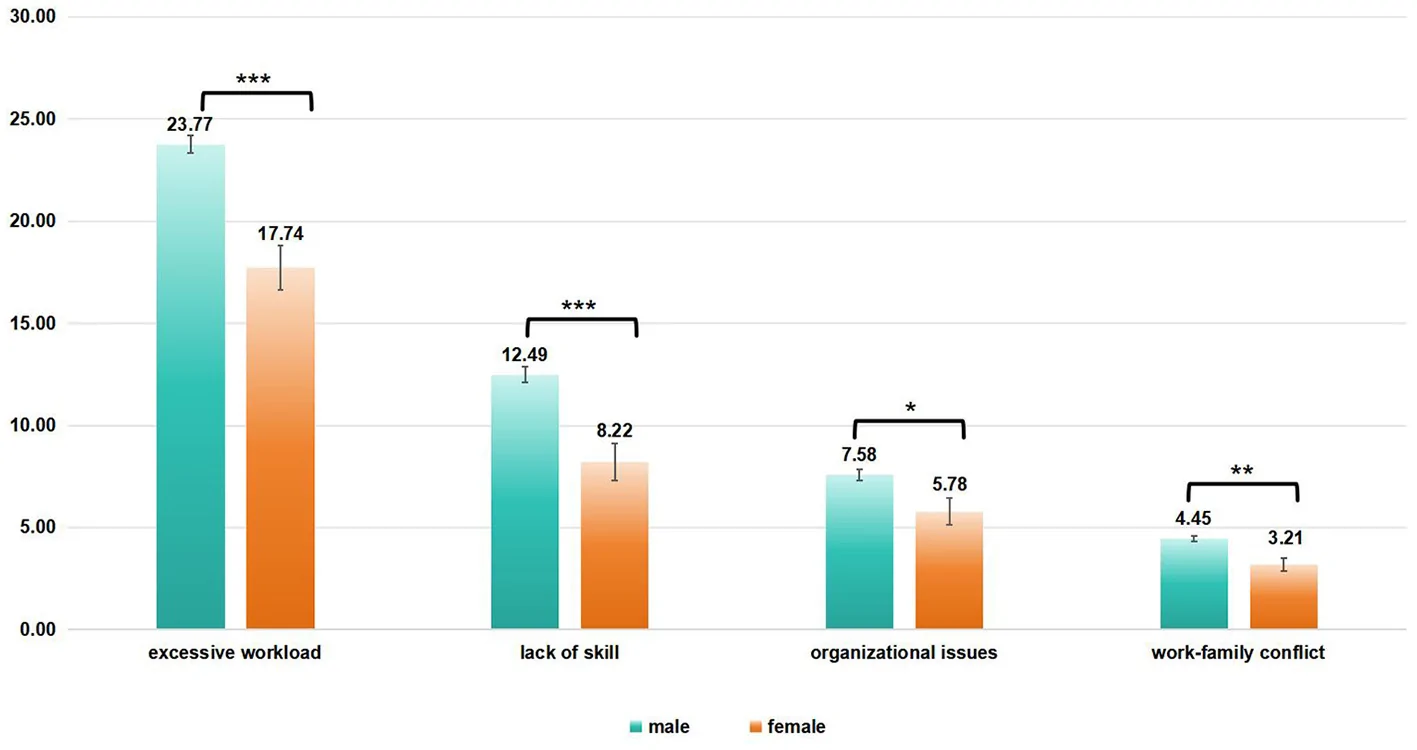
Factor scores of CPSPSS-R for male police officers and female police officers. Error bars reflect standard error of mean. ***p < 0.001, **p < 0.01, *p < 0.05.

**Figure 2:**
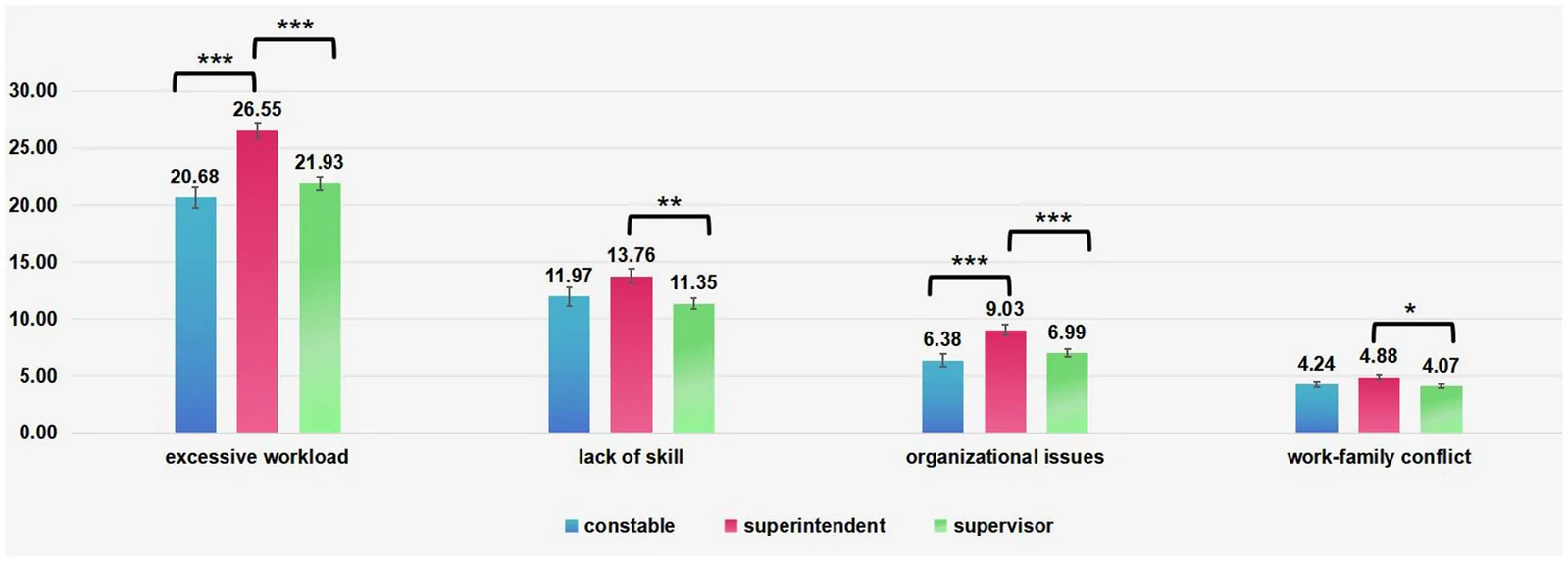
Factor scores of CPSPSS-R for three ranks of public security police officers. Error bars reflect standard error of mean. ***p < 0.001, **p < 0.01, *p < 0.05.

An independent *t*-test revealed significant differences between genders for all four factors of CPSPSS-R (Figure 1), and found that stress experienced by male police officers was significantly higher than their female counterparts (Table 10). In addition, the ANOVA showed significant differences among ranks (Figure 2). Multiple comparisons with Bonferroni correction showed that superintendents experienced significantly more stressors than officers of the other two ranks. Detailed comparisons are presented in Table 11.

**Table 10:** Gender differences of stressors in public security police officers.

Factors	Male		Female		*t*	*p*	Cohen's *d*
	Mean	SD	Mean	SD			
Excessive workload	23.77	13.80	17.74	12.10	5.099	< 0.001	0.46
Lack of skill	12.49	11.65	8.22	9.92	4.389	< 0.001	0.39
Organizational issues	7.58	8.31	5.78	7.29	2.515	0.013	0.23
Work-family conflict	4.45	4.06	3.21	3.55	3.591	0.001	0.33
CPSPSS-R	48.29	33.90	34.95	29.66	4.601	< 0.001	0.42

**Table 11:** Stressors differences among ranks in public security police officers.

Factors	Constable		Superintendent		Supervisor		*F*	*p*	$\eta_{\text{p}}^{2}$
	Mean	SD	Mean	SD	Mean	SD			
Excessive workload	20.68	14.06	26.55	13.95	21.93	13.25	17.592	< 0.001	0.030
Lack of skill	11.97	12.66	13.76	12.13	11.35	11.08	4.624	0.010	0.008
Organizational issues	6.38	8.29	9.03	9.05	6.99	7.75	9.467	< 0.001	0.016
Work-family conflict	4.24	4.19	4.88	4.08	4.07	3.95	4.48	0.012	0.008
CPSPSS-R	43.26	35.43	54.22	35.05	44.33	32.46	11.382	< 0.001	0.020

## 4 Discussion

This study aimed to develop and validate a revised version of the PSS. The resulting CPSPSS-R is an essential tool for assessing stressors, specifically among Chinese public security police officers. The CPSPSS-R demonstrated excellent reliability and validity, establishing it as a robust instrument for measuring stressors in this population.

This study makes three significant contributions to the field. First, it provides an updated and culturally relevant tool for assessing stressors among Chinese public security police officers while also addressing the growing need for research on stress management and well-being among police officers (1, 3, 5, 10). Second, the psychometric evaluation of CPSPSS-R identified four key dimensions of stressors experienced by public security police officers: *excessive workload, lack of skill, organizational issues and work-family conflict*. These findings provide empirical evidence to support improvements in police management systems aimed at reducing these stressors. Third, a preliminary investigation with CPSPSS-R revealed associations between stressors and various demographic variables among public security police officers in mainland China. This finding highlights the need for targeted interventions tailored to different subgroups within the police force.

### 4.1 Psychometric characteristics and dimensions of CPSPSS-R

Compared to previous scales such as the PSS and the OSSPPSO, the CPSPSS-R demonstrates notable improvements in both psychometric properties and practical application (4, 31).

On the one hand, while the PSS provides a broad overview of stressors across different police departments, it lacks the specificity for public security officers and includes outdated items (31). The CPSPSS-R addresses these limitations by refining the target population and incorporating relevant modern-day stressors, such as social media and economic pressures. Furthermore, the CPSPSS-R comprises fewer items while maintaining higher reliability, further strengthening its advantages over the PSS. On the other hand, the OSSPPSO does focus on public security officers. However, it overlooks the work-home interface, a critical stressor in high-pressure jobs [(4, 38, 39); Yang et al., 2023]. The CPSPSS-R explicitly includes items on family-related difficulties, recognizing the impact of work-home balance on officers' wellbeing.

Interestingly, one item on work-home balance (“Infrequent communication with family due to long work hours”), which was originally categorized under the dimension *interpersonal activities* in the PSS (31), was validated in this study as an indicator of the *excessive workload* factor. Similar reclassifications have been observed in previous studies of occupational stress. For example, Williams and Copper (1998) found that the item “demands my work makes on my relationship with my partner or children” was classified under the *workload* dimension rather than *work-home balance*. This suggests that such item primarily reflect the source of stress—heavy work demands—rather than the family itself.

The four CPSPSS-R dimensions provide valuable insights into the unique challenges faced by public security police officers in China. The dimension of *excessive workload* underscores the need for better workload distribution and resource management to prevent burnout and overwork. Workload-related stressors is a well-documented issue faced by public security police (1, 5, 10, 13; Chen et al., 2022). Persistent excessive overtime and emergency standby duties continuously deplete police officers' psychological coping resources, exhausting emotional regulatory capacity and eroding stress adaptation over time. From a psychological adaptation perspective, this dimension captures the most pervasive environmental stressor that disrupts stable mental functioning in high-demand police work. To address this, measures such as increasing police recruitment, expanding staff, and redistributing workloads to ensure even deployment should be considered.

The *lack of skill* dimension highlights the need for ongoing training and professional development to help officers adapt to evolving job demands. Particularly, police training should emphasize adaptability to public scrutiny, especially given the increasing influence of social media and public opinion.

The *organizational issues* dimension emphasizes the importance of fostering a more supportive work environment and reducing bureaucratic inefficiencies. Issues related to organizational management in police forces have been widely discussed in both Western and Asian contexts (52–55). Given that policing involves hierarchical power structures and demands strict obedience in police work, it is essential to combat corruption and nepotism while encouraging fairness in promotions and career advancement.

Consistent with the work-home resources model (39), the *work-family conflict* dimension reflects cross-domain resource depletion that impairs police officers' holistic psychological adaptation. Unresolved interrole strain undermines balanced life adjustment and produces negative correlation with life satisfaction. This dimension underscores the critical value of work-home balance and institutional family-friendly measures including flexible scheduling and family support programs; such policies act as vital adaptive interventions to replenish exhausted personal psychological resources, mitigate work-family stressors, and boost officers' overall job satisfaction while safeguarding their long-term mental wellbeing amid chronic occupational pressure.

### 4.2 Correlations between stressors and demographic variables

Our analysis revealed significant correlations between stressors and demographic variables. Younger police officers and those with less experience reported higher levels of stressors, likely due to their relative inexperience and limited coping mechanisms. Interestingly, police officers with higher education levels also reported increased stress. There are two possible explanations for this. First, since 1999, higher education has been widely popularized in mainland China, with gross university enrollment rate reaching 60.2% in 2023 (56, 57). As a result, younger police officers are more likely to have higher academic qualifications than previous generations. Second, higher educational levels may be associated with greater responsibilities and expectations, leading to increased job-related stressors. These findings highlight the need for targeted stress management interventions for different demographic groups. Support programs should be designed to assist younger, less experienced officers in developing coping strategies, while also addressing the needs of those with higher qualifications who face increased job demands.

Significant gender differences were observed, with male officers reporting higher stress levels across all factors. This may be partly attributed to traditional gender roles and masculinity norms in East Asian societies (58), where men are expected to achieve great career success (59). For instance, in civil service roles such as public security policing, East Asian families often expect men to attain higher-ranking positions, as this is associated with family honor is seen as a precondition for being a “good husband” and “good son” (60). These societal pressures may contribute to heightened stress levels among male police officers. However, when it comes to the gender differences in stressors, we should be very cautious in interpreting them. While our study suggests that gender differences are not significantly related to police rank, it is possible that they are related to characteristics of specific positions. In China, for example, female police officers are more likely to have administrative roles than frontline duties (Li et al., 2016). This requires further research and verification.

Rank-based stress disparities were also evident, with superintendents experiencing higher stress levels than constables and supervisors. This finding contrasts with previous studies on occupational stress and burnout in police officers that suggest that low-ranking policemen experience greater work-related stress (61, 62). A possible explanation for this discrepancy is that the superintendents face combined pressures of managerial duties and front-line responsibilities. Additionally, their work involves navigating bureaucratic red tape, administrative matters, frequent policy and legislation changes, and work assignment responsibilities. They must juggle these responsibilities but without the autonomy of rule-making (as seen with supervisors) or the singular focus of executing orders (as with constables). These findings emphasize the importance of developing rank-specific stress management strategies, including targeted counseling and leadership development programs for public security police officers. Such interventions can help cope with stressors across different hierarchical levels within the public security police force.

### 4.3 Limitations and directions for future research

Although the CPSPSS-R marks an important advancement in measuring stressors among Chinese public security police officers, its limitations need to be acknowledged. First, the study relied on self-reported data, which is vulnerable to response biases such as social desirability or underreporting of stress. Future research could incorporate other measures of stress, such as physiological indicators or observer evaluations, to complement self-reported data. Second, while the CPSPSS-R focuses on stressors, it does not account for coping mechanisms or resilience—factors that are crucial to understand how officers manage stress. Future research should explore the interaction between stressors and coping strategies to inform more comprehensive stress management interventions. Third, this study's cross-sectional design limits causal inferences regarding the relationship between stressors and demographic variables. Longitudinal studies are needed to track how stressors evolve over time and their long-term impact on officers' mental health and job performance.

## 5 Conclusion

The CPSPSS-R represents an important advancement in assessing stressors among public security police officers in China. By identifying four key dimensions—*excessive workload, lack of skill, organizational issues* and *work-family conflict*—the CPSPSS-R offers a comprehensive and culturally adaptive tool for understanding the unique challenges faced by this population. The scale's strong psychometric properties and ability to capture modern stressors, such as social media and economic pressures, make it a valuable resource for both researchers and practitioners. Significant correlations between stressor levels and demographic indicators, alongside pronounced subgroup disparities in stress exposure, expose differentiated vulnerability profiles in public security police officers' psychological adjustment, which underscores the urgency of stratified, stressor-specific mental health interventions to cultivate adaptive resilience and sustain long-term mental wellbeing for officers working within chronically demanding, high-risk law enforcement environments. Beyond facilitating evidence-based, tailored stress management strategies that jointly promote police officers' occupational performance and holistic wellbeing, the CPSPSS-R opens avenues for future integrated research: subsequent studies may combine this scale with assessments of resilience, coping styles, and clinical mental health outcomes to generate a holistic picture of psychological adaptation trajectories amid enduring occupational challenges.

## Data Availability

The raw data supporting the conclusions of this article will be made available by the authors, without undue reservation.
