# Supplementary material for: Validating and applying a multidimensional scale to measure public security police stressors in China
Source: Front Public Health. 2026 Sep 3;14:1932210. doi: 10.3389/fpubh.2026.1932210 (PMC13582256; doi:10.3389/fpubh.2026.1932210)
Supplement: Supplementary file 1 [file Data_Sheet_1.pdf]

Appendix 1

Revised Chinese Public Security Police Stressors Scale (CPSPSS-R)

Instructions: Below is a list of situations and events that police officers working in public safety may encounter. Please indicate whether you have experienced these situations in the past six months and, if so, indicate to what extent this event impacted you mentally.

No.	Event	Have not experienced	Have experienced (Please indicate the impact level)				
			No Impact	Mild	Moderate	Severe	Extreme
1	Frequent overtime work affecting regular rest						
2	Heavy workload with demanding tasks						
3	Excessive non-policing duties						
4	Increased responsibility due to accountability systems						
5	Complicated case-handling procedures						
6	Excessive on-duty responsibilities						
7	Irregular working hours						
8	Disproportionate pay relative to work effort						
9	Disproportionate personnel deployment; lack of frontline officers						
10	Infrequent communication with family due to long work hours						
11	High societal expectations leading to a lack of respect and understanding						
12	Insufficient income causing financial strain						
13	Family's lack of understanding of police work						
14	Erosion of family's trust due to job demands						
15	Bringing work-related emotions home, causing family conflicts						
16	Conflicts among leaders affecting subordinates' work						
17	Lack of recognition from superiors						
18	Leaders assigning tasks without regard for subordinates' circumstances						
19	Bureaucratic rigidity in leadership practices						

20	Favoritism or inconsistent treatment by superiors						
21	Leadership emphasis on discipline over positive feedback						
22	High-pressure competition among colleagues						
23	Nepotism affecting case handling						
24	Lack of proficiency in job skills						
25	New job requirements exceeding current knowledge						
26	Lack of self-regulation and coping abilities						
27	Difficulty communicating and integrating with colleagues						
28	Poor social skills						
29	Pessimistic attitude toward personal mental health						
30	High job-related danger and concerns for personal safety						
31	Monotonous and dull work content causing irritability						
32	Worry about failing to complete case tasks						
33	Frequent inspections, evaluations, and assessments						
34	Coping with public scrutiny through photographs or live-streams during law enforcement						
35	Public security threats due to emergency incidents						
36	Criticism or disapproval from colleagues						
37	Fear of public opinion and negative comments						
